# Flashforward imagery in speech anxiety: Characteristics and associations with anxiety and avoidance

**DOI:** 10.3389/fpsyg.2022.975374

**Published:** 2022-10-04

**Authors:** Marjolein R. Thunnissen, Maaike H. Nauta, Peter J. de Jong, Marleen M. Rijkeboer, Marisol J. Voncken

**Affiliations:** ^1^Department of Clinical Psychology and Experimental Psychopathology, University of Groningen, Groningen, Netherlands; ^2^Accare, University Center for Child and Adolescent Psychiatry, Groningen, Netherlands; ^3^Department of Clinical Psychological Science, Maastricht University, Maastricht, Netherlands

**Keywords:** speech anxiety, imagery, flashforwards, social anxiety, cognitive processes

## Abstract

Speech anxiety (SA) is a highly prevalent social fear. Prospective ‘flashforward’ (FF) imagery of an upcoming social catastrophe may be a particularly important cognitive factor in SA persistence via eliciting anxiety and avoidance behaviors. Since earlier research on imagery and social anxiety has not strictly differentiated between types of negative imagery, the occurrence, precise features, and impact of FF imagery remain unclear. We therefore examined the phenomenological characteristics of FF imagery in SA and mapped the relationship between FF imagery features and anxiety and avoidance. Female participants who approached clinical levels of SA (*N* = 60) completed questionnaires on SA and avoidance behaviors, and rated anxiety and avoidance in anticipation of an actual speech. FF imagery and emotionally linked autobiographical memories were assessed with semi-structured interviews. All participants reported recurring FF images, which were experienced as vivid, distressing, field perspective images with accompanying negative feelings. Image distress and feelings of threat showed most consistent associations with SA and avoidance measures. Findings add to the conceptualization of SA, and support the clinical relevance of assessing FF imagery. Future experimental studies on FF imagery characteristics are necessary to test the proposed causal impact in SA persistence and to inform additional treatment targets.

## Introduction

Speech anxiety (SA) is a highly prevalent social fear; fear of public speaking was reported as the most common lifetime social fear with prevalence rates reaching 21.2% in a national survey in the United States (Ruscio et al., 2008). The core cognitive concern in SA is similar to that in social anxiety disorder (SAD): being scrutinized and judged negatively by others (Bögels et al., 2010). According to dominant models of SAD, negative mental images play an important role in the maintenance of anxiety (e.g., Clark and Wells, 1995; Heimberg et al., 2010). Mental imagery abnormalities are not only involved in models of SAD but are also deemed relevant across emotional disorders (Holmes and Mathews, 2010). For example, in post-traumatic stress disorder, negative intrusive imagery in the form of trauma memories plays a pivotal role (Ehlers and Clark, 2000), and in depression, negative intrusive imagery and impoverished positive imagery seem to be relevant (Holmes et al., 2016). In social anxiety, it has been suggested that in (impending) social situations, negative distorted images that display one’s public self are activated, as these situations are perceived as dangerous for being rejected by possibly behaving inappropriately (Clark and Wells, 1995). Along with detailed monitoring of how one may come across, negative images proposedly increase perceived social danger and anxiety symptoms, as well as the use of safety behaviors (such as speaking softly or rehearsing excessively) to avoid rejection.

In line with the theoretical models of SAD, empirical research has shown that individuals with SAD indeed report to experience spontaneously occurring recurrent negative images (e.g., Hackmann et al., 2000). These distorted images are experienced as happening in the ‘here and now’ and being a true representation of how one appears to others. The content of the imagery typically represents the main fears (e.g., performing badly or being negatively evaluated; Hirsch and Holmes, 2007) and is often found to be linked to a particular autobiographical memory (Hackmann et al., 2000; Schreiber and Steil, 2013). Negative images occur more frequently in individuals with SAD than in individuals without SAD, are rated as more vivid and distressing, and have been found to be more often seen from an observer than from a field perspective (Hackmann et al., 1998; Schreiber and Steil, 2013). In analogue samples of high versus low socially anxious individuals, findings on imagery characteristics have been less consistent (Moscovitch et al., 2011; Ashbaugh et al., 2019). Yet, when instructed to hold a negative versus a neutral image in mind during social interaction or a speech, higher levels of anxiety, more safety behaviors, and worse performance were reported in both SAD groups and non-SAD comparison groups (Hirsch et al., 2003, 2004, 2006). Engaging in anticipatory processing (involving imagery of potential catastrophes) versus distraction before a speech showed similar effects on anxiety (Hinrichsen and Clark, 2003).

Thus far, research on imagery and social anxiety did not strictly differentiate between types of negative imagery. Imagery definitions and assessments have varied and the time orientation of relevant intrusive imagery has not always been reported (Brewin et al., 2010; for observational studies in analogue samples that did report time orientation, see Homer and Deeprose, 2017; Ashbaugh et al., 2019). Yet, prospective or future-oriented negative imagery may be of particular importance in the persistence of SA and, therefore, may be a relevant type of imagery to specifically assess. Prospective imagery of potential or future threats seems to have an anticipatory and preparatory function, and there is evidence that such imagery may cause and inflate anxiety and avoidance behaviors related to a(n) (upcoming) situation (Mertens et al., 2020). Findings that imagery is associated with increased subjective probability of the imagined outcome and prompted future behavior (Holmes and Mathews, 2010) further underline its potential impact. Thus, generating or experiencing such negative ‘flashforward’ (FF) imagery related to a social situation, depicting the expectation of feared outcome (e.g., rejection by others), might especially fuel perceived danger and feelings of imminent threat, which in turn may increase anxiety and safety behaviors that impede correction of dysfunctional beliefs and biased expectancies (Clark and Wells, 1995). In line with this, some studies focused on targeting FF imagery in the context of SA to degrade these images (e.g., Engelhard et al., 2012). However, the occurrence, precise features, and impact of FF imagery in social anxiety remain unclear. Therefore, in the current study, we specifically focused on mapping FF imagery in SA and aimed to improve insight in the relevant features of FF imagery and how these features relate to anxiety and avoidance, including safety behaviors.

First, we examined to what extent FF images are experienced in (impending) speech situations in SA, and whether FF images are characterized as being vivid, distressing, accompanied by negative feelings, and seen from an observer perspective. Also, we examined the similarity of FF imagery to associated memories to further explore how to categorize these images (Çili and Stopa, 2021). Imagined catastrophes that might currently elicit anxiety and avoidance behaviors in SA, may be based on or linked to memories of past fearful events, as was also found in studies with broad assessments of negative imagery in social anxiety (Hackmann et al., 2000; Moscovitch et al., 2011; Schreiber and Steil, 2013). In patients with severe health anxiety, for example, it was found that even though the majority of patients reported future-related intrusive images, many of these images concerned a (distorted) memory of an earlier event or were associated with a memory (Muse et al., 2010). This fits with research implying that future and past thinking rely on similar cognitive processes and neural circuitry, and that the past is proposedly used to simulate prospective events (Schacter et al., 2007). In sum, as our first aim, we wanted to assess whether individuals with SA report FF imagery that they experience recurrently in their daily lives, and to describe the features of their FF imagery.

Second, if FF imagery is indeed important in the persistence of SA, FF imagery should be associated with anxiety and avoidance. Therefore, we evaluated the associations between reports of SA and avoidance behaviors, including safety behaviors, and FF image vividness, distress, and perspective. Additionally, we explored the relationship between SA and avoidance behaviors and feelings that something terrible will happen concomitant to FF imagery, which may indicate perceived danger or probability of negative outcome; this characteristic has also been found to differentiate patients with anxiety disorders from non-anxious individuals (Morina et al., 2011). To increase validity of information on the relationship between these FF imagery features and anxiety and avoidance, we included reports related to a social-evaluative threat induction (i.e., a speech), since cognitive concerns and related processes are proposed to be activated in anticipation of, during, and after fearful events (Clark and Wells, 1995). We focused on reports of anticipatory anxiety and the urge to avoid this upcoming threatening situation, given that negative affect is found to be highest in this phase (Van Boven and Ashworth, 2007), and negative images frequently occur while anticipating an upcoming event (Chiupka et al., 2012). In sum, as our second aim, we wanted to map in more detail whether and to what extent FF imagery characteristics are associated with SA and avoidance behaviors.

In short, this study aimed to increase insight in the specific characteristics of FF imagery and their associations with anxiety and avoidance. This may help improve the conceptualization of SA, and may point to relevant clinical implications for SA assessment and treatment. The current study therefore examined FF imagery in the context of SA (i.e., prospective imagery of feared catastrophe related to SA) with an imagery interview, and investigated how features of FF imagery related to SA and avoidance behaviors as assessed with retrospective questionnaires as well as with anxiety and avoidance ratings in anticipation of an actual speech task.

## Materials and methods

### Participants

The study sample consisted of 60 female students (*M* age = 21.6, *SD* = 2.6) who approached clinical levels of SA in a screening. Participants were recruited using flyers and posters at the University of Maastricht, as well as online platforms intended for participation in scientific research. Students were asked to respond if they identified themselves as speech anxious. To make sure that participants experienced considerable levels of SA as well as interference, we used the following inclusion criteria in a screening: (i) a score of 16 or higher on the Personal Report of Confidence as a Speaker (PRCS) (Paul, 1966) and (ii) a score of 3 or higher on the Fear Questionnaire for public speaking anxiety (FQ-PS) (Dutch versions). On the PRCS, which consists of 30 “true” or “false” statements measuring confidence in speaking ability, higher scores represent less confidence. To prevent inclusion of students that just do not like to give presentations, the following exclusion criterion was used: responding positively to item 17 (“Although I do not enjoy speaking in public, I do not particularly dread it”). The original FQ by Marks and Mathews (1979) was adapted to include one item measuring anxiety in public speaking situations, ranging from 0 (“I feel completely comfortable”) to 8 (“I am in total panic”). The FQ-PS was added after the first weeks of data-collection; therefore, the first study participants were screened using only the PRCS.

### Measures

#### Baseline sample characteristics

Participants received questions about demographics and completed the following measures to assess sample characteristics.

To include an assessment of general imagery ability, a Dutch translation of the Questionnaire upon Mental Imagery (QMI; Sheehan, 1967) was used to assess mental imagery vividness. The QMI consists of five items for each of the seven senses, resulting in 35 items in total. Participants are asked to imagine and rate the items on a 7-point scale ranging from 1 (“as perfectly clear and vivid as in reality”) to 7 (“I think about it, but I cannot imagine it”), with lower scores representing a greater ability to visualize. Previous studies have reported high internal consistency (Cronbach’s alpha = 0.88; Lee and Kwon, 2013). In this sample, Cronbach’s alpha was .91.

The Center for Epidemiologic Studies Depression Scale (CES-D; Bouma et al., 1995) was used to assess depressive symptoms as a means to provide a more comprehensive description of the current sample. Symptoms of social anxiety and depression often show comorbidity (e.g., Moscovitch et al., 2005). This self-report questionnaire consists of 20 items about the frequency of symptoms in the past week. Items are rated on a 4-point scale ranging from 0 (“rarely or never”) to 3 (“mostly or always”). A score of 16 or higher is considered as ‘possible depression’. Good internal consistency has been reported (0.79–0.92; Bouma et al., 1995), as well as good construct validity. In the current sample, Cronbach’s alpha was .90.

Finally, Dutch translations of the Social Phobia Scale (SPS) and the Social Interaction Anxiety Scale (SIAS) (Mattick and Clarke, 1998) were used to assess fears of scrutiny during routine activities such talking in public and fears of more generalized social interaction, respectively. Both contain 20 statements that are rated on a 5-point scale ranging from 0 (“not at all characteristic or true for me”) to 4 (“extremely characteristic or true for me”), with higher scores representing higher social fears. The scales showed high internal consistency (Cronbach’s alpha = 0.94; Mattick and Clarke, 1998), and good construct and discriminant validity. In this sample, Cronbach’s alpha of the SPS was .91 and of the SIAS .92.

#### Anxiety and avoidance measures

We used two retrospective questionnaires to assess SA and avoidance behaviors. To assess global levels of SA, the PRCS (Paul, 1966) was also completed after inclusion in the study. The original questionnaire demonstrated high internal consistency (Cronbach’s alpha = 0.91) and adequate convergent validity (Daly, 1978). Cronbach’s alpha in the current sample was 0.76. To assess avoidance behaviors in speech situations, an adapted and translated version of the Subtle Avoidance Frequency Examination (SAFE; Cuming et al., 2009) was used. Items were selected and adjusted to apply to speech situations, resulting in 30 items of possible strategies used rated on a 5-point scale from 1 (“never”) to 5 (“always”), with higher scores representing more safety behaviors. High internal consistency of the original scale has been found (Cronbach’s alpha = 0.91; Cuming et al., 2009), as well as good discriminant and construct validity. Cronbach’s alpha in this sample was .80.

In addition, we used two rating scales to assess anticipatory anxiety and avoidance related to an upcoming speech. Participants rated the following two items on a scale from 0 (“not at all’) to 100 (“extremely”): “How anxious do you feel, now you know that you are going to give a speech?” and “What is your urge to avoid the upcoming speech?”

#### Flashforward imagery interview

The semi-structured imagery interview developed by Hackmann et al. (2000) was translated and adapted for the current study, providing a specific focus on prospective imagery of catastrophic scenarios related to SA, and on autobiographical memories associated with such FF imagery. A previous adaptation of this imagery interview for a study on body dysmorphic disorder showed adequate test–retest reliability (Osman et al., 2004). In the current interview, after explaining what an FF image is (“an impression or picture of a catastrophic scenario that could take place”), participants were asked whether they recognize having such FF images or impressions “when their SA is really bad” and if so, to describe their FF image. Simultaneously, the interviewer checked whether this image recurrently occurred when participants experience SA. Participants were asked to close their eyes and received probes when describing their FF image (e.g., “What do you see? Who are with you? What is happening? What do you feel? How does the situation develop? Is this the catastrophic scenario? Is this the worst thing that could happen?”) The interviewer then asked participants to rate the image on the following aspects: having the qualities of a clear visual image (“yes,” “probably,” or “no”); image is experienced like a… (“snapshot,” “series of pictures” or “film”); perspective (ranging from, −2, “totally from my own eyes,” to 2, “totally from observers’ eyes”); vividness, realness, and distress (ranging from 0, “not at all,” to 100, “extremely”); and concomitant feelings of anxiety, anger, sadness, guilt, shame, helplessness, and that something terrible will happen (ranging from 1, “not at all,” to 5, “extremely”). Next, participants were asked to relive the most anxiety-provoking part of the FF and focus on the emotions experienced; emotion is thought to be an important link between prospective thoughts and autobiographical memories (Demblon and D’Argembeau, 2016). Then, they were instructed to let the image fade away and see whether a memory from their childhood came back (a so-called affect bridge, which is often used in imagery rescripting). If so, they were asked to describe the memory. They rated similarity of the FF image and memory (ranging from 0, “not at all,” to 100, “extremely). The interview lasted approximately 25–30 min.

To assess whether participants had indeed indicated to recurrently experience the reported FF image in their daily lives, recurrence of FF imagery was coded based on the interview recordings. A category of either “recurrent” (1) or “not recurrent” (0) was assigned, depending on whether the participant expressed that she did or did not experience (key parts of) the FF image recurrently in speech situations (“recurrent” included clearly and probably/partly recurrent).

### Procedure

The current study considered data of the first session of a larger study in which also other measures were completed that are beyond the scope of this study. After informed consent, this first session started with participants completing the measures on baseline sample characteristics and the questionnaires on global levels of SA and avoidance behaviors on a computer. Other measures that were completed included questionnaires on cognitive factors such as phobic beliefs and self-focused attention, and an interview on social phobia. Then, participants were instructed about an upcoming speech (social-evaluative threat induction) and completed the ratings on anticipatory anxiety and avoidance. Next, participants blindly selected a speech topic from a box with cards listing topics pre-selected by the researchers (‘impromptu speech’). They gave a five-minute speech in a room with two observers (trained confederates) as the audience. After the speech, participants again completed other measures including part of the baseline questionnaires. Then, the imagery interview was administered on FF imagery related to SA in their daily lives. After the interview, participants proceeded with the remainder of the study (including randomization to a condition and taking part in an imagery rescripting or control procedure, followed by post-intervention assessments), which is not further described here.

### Statistical analyses

Analyses were performed using IBM SPSS Statistics 25. First, we computed means and SDs or frequencies of FF imagery characteristics, recurrence, and similarity to associated memories. Second, we computed correlations between the FF imagery characteristics of our primary focus (vividness, distress, perspective, and the feeling that something terrible will happen) and the anxiety and avoidance measures. We mapped the effect strengths of these associations and chose to make no corrections for multiple testing in this phase to prevent an increased type II error rate.

## Results

### Sample characteristics and anxiety and avoidance measures

Means and SDs or frequencies for the screening measures, baseline sample characteristics, and anxiety and avoidance measures can be found in Table 1. Participants with a non-Dutch nationality did have Dutch language proficiency. The FQ-PS screening had not yet been administered to four participants, who were among the first study participants (also see Method); they were included using only the PRCS screening.

**Table 1:** Descriptives for screening measures, baseline sample characteristics, and anxiety and avoidance measures.

	Mean (*SD*)/*N* (%)	Range
*Screening measures and baseline sample characteristics*		
Age	21.57 (2.64)	18–29
Nationality (*n* (%) Dutch)	52 (86.7)	
Education (*n* (%) psychology, health sciences, medicine, other)	24 (40.0), 17 (28.3), 12 (20.0), 7 (11.7)	
FQ-PS screening (*n* = 56)	4.87 (1.25)	3–7
PRCS screening	24.02 (3.40)	16–30
QMI	92.37 (21.80)	42–148
CES-D	12.83 (8.27)	1–36
SPS	25.50 (13.36)	1–64
SIAS	30.50 (14.48)	7–70
*Anxiety and avoidance measures*		
PRCS – trait speech anxiety	23.38 (3.63)	13–28
SAFE – trait speech avoidance	85.02 (12.97)	52–117
State anticipatory anxiety	68.75 (14.57)	30–100
State anticipatory avoidance	55.17 (30.17)	0–100

FQ, Fear Questionnaire Speech Anxiety; PRCS, Personal Report of Confidence as a Speaker; QMI, Questionnaire upon Mental Imagery; CES-D, Center for Epidemiologic Studies Depression Scale; SPS, Social Phobia Scale; SIAS, Social Interaction Anxiety Scale; SAFE, Subtle Avoidance Frequency Examination questionnaire.

### Flashforward imagery characteristics, recurrence, and similarity to associated memories

All 60 participants reported an FF image related to their SA. In Table 2, examples of reported FF imagery are provided.

**Table 2:** Examples of reported flashforward imagery and associated memories.

FF imagery	
Example 1	‘… I imagine how others will judge me *(negatively)*. I see how, afterwards *(after the speech situation)*, the others are talking about how it was … I think I could have done better or could have said something else… the catastrophic scenario is that they will form a negative image about me after the situation…’
Example 2	‘… I prepare to start my presentation, and at that moment everyone starts looking at me and is waiting for me to say something… I am tense… in the catastrophic scenario, right at the first word I start to stutter so severely that it takes me a long time to start again, and people look away or do not respond… in the worst case scenario I become really self-conscious, which only increases the pressure, and I may not be able to pick it up again… I am afraid that the others may find me pathetic or incompetent… that they judge me *(negatively)*’
**Associated memory**	
Example 1	‘… I am 10 years old… in the train in Italy… I am afraid that either me or the rest of my family will get off the train and that I will be left alone at the station or inside the train… nothing actually happens… but I can remember that I was really scared’
Example 2	‘… I was in the second grade, 13 years old, in a new class… 30 other people, all faced to the front of the classroom… they asked us to introduce ourselves, and when it was my turn, I stuttered when I said my name… people laughed… one boy repeated my name in the same stuttering manner… I feel emotional…’

FF = Flashforward.

Means and SDs or frequencies for the FF imagery characteristics, recurrence, and similarity to associated memories can be found in Table 3. On average, FF images were rated as rather clear, vivid, distressing, and real, and specifically elicited feelings of shame, helplessness, and anxiety. Moreover, FF images were – grosso modo – either perceived as a film or a snapshot, and, roughly half of the participants rated their FF image as having an explicit field perspective (*n* = 31, 51.7%), whereas about one third rated their image as having an evident observer perspective (*n* = 20, 33.3%). On average, FF’s were perceived more from a field than from an observer perspective.

**Table 3:** Flashforward imagery characteristics, recurrence, and similarity to associated memories.

	Mean (*SD*)/*N* (%)	Range
*FF imagery*		
Clear visual image (*n* (%) yes, probably, no)	36 (60), 19 (31.7), 5 (8.3)	
Image is like a… (*n* (%) snapshot, series of pictures, film)	22 (36.7), 10 (16.7), 28 (46.7)	
Perspective^1^	−0.43 (1.84)	−2 - +2
Vividness	69.75 (18.10)	30–100
Realness	61.17 (23.37)	10–100
Distress	65.75 (22.83)	0–100
Anxiety	3.30 (1.17)	1–5
Anger	1.85 (1.06)	1–5
Sadness	2.53 (1.36)	1–5
Guilt	1.82 (1.02)	1–5
Shame	3.53 (1.11)	2–5
Helplessness (*n* = 59)	3.31 (1.24)	1–5
The feeling that something terrible will happen	2.67 (1.30)	1–5
Recurrence (*n* = 53) [*n* (%) recurrent]	53 (100)	
Associated memory (*n* = 59) [*n* (%) with associated memory]	57 (97)	
Similarity FF image – memory (*n* = 57)	56.32 (24.25)	0–100

^1^Perspective ranged from, −2, “totally from my own eyes,” to 2, “totally from observers’ eyes.” FF, Flashforward.

All participants of whom the required information was available (*n* = 53) indeed indicated that they recurrently experienced FF imagery. The required information for checking recurrence was missing for seven participants due to one missing audio file and six interview recordings with no explicit (follow-up) questions on recurrence by the interviewer. Furthermore, of those who completed the memory section of the interview (*n* = 59), almost all (*n* = 57, 97%) reported a (specific) memory associated with their FF image; yet, the similarity of both was rated as moderate. One participant had not completed the memory section of the interview, and two participants were not able to report on an associated (specific) memory. See Example 1 and 2 in Table 2 for illustrations of associated memories that were rated as not at all similar and as quite similar to the FF image, respectively.

### Associations between flashforward imagery characteristics and anxiety and avoidance

Correlations between the FF imagery characteristics of our primary focus and the anxiety and avoidance measures can be found in Table 4. Spearman correlations were computed for correlations including ordinal variables as well as the PRCS. Other variables sufficiently met the statistical assumptions for using Pearson correlation.

**Table 4:** Correlations between flashforward imagery characteristics and anxiety and avoidance measures.

	Imagery characteristics				Anxiety and avoidance			
	Perspective^1,2^	Vividness	Distress	Feeling TER^1^	PRCS^1^–SA	SAFE–avoid	Anticip -anx	Anticip -avoid
*Imagery characteristics*								
Perspective^1,2^	1	−0.07	0.20	0.20	−0.04	0.34^**^	0.08	−0.02
Vividness	−	1	0.43^**^	0.39^**^	0.34^**^	0.35^**^	0.04	0.13
Distress	−	−	1	0.66^**^	0.35^**^	0.45^**^	0.32^*^	0.30^*^
Feeling TER^1^	−	−	−	1	0.42^**^	0.51^**^	0.36^**^	0.32^*^
*Anxiety and avoidance*								
PRCS^1^–SA	−	−	−	−	1	0.28^*^	0.33^*^	0.34^**^
SAFE–avoid	−	−	−	−	−	1	0.37^**^	0.31^*^
Anticip-anx	−	−	−	−	−	−	1	0.55^**^
Anticip-avoid	−	−	−	−	−	−	−	1

^1^Spearman’s rho, other correlation coefficients are Pearson correlations. ^2^Perspective ranged from, −2, “totally from my own eyes,” to 2, “totally from observers’ eyes.” PRCS, Personal Report of Confidence as a Speaker; SA, speech anxiety; SAFE, Subtle Avoidance Frequency Examination questionnaire; avoid, avoidance behaviors; Anticip-anx, anticipatory anxiety; Anticip-avoid, anticipatory avoidance; Feeling TER, ‘the feeling that something terrible will happen’.

* *p* < 0.05;

** *p* < 0.01.

Perspective of FF imagery showed a moderate correlation with avoidance behaviors (more evident observer perspective was related to more avoidance behaviors) but very weak and non-significant correlations with global levels of SA or anticipatory anxiety and avoidance as reported just before the speech task. FF imagery vividness was moderately related to global SA levels and avoidance behaviors but weakly (n.s.) to anticipatory anxiety and avoidance. FF imagery distress showed moderate correlations with all four anxiety and avoidance measures. Finally, the feeling that something terrible will happen concomitant to FF imagery was moderately to strongly related to all four anxiety and avoidance measures.

## Discussion

This study examined the characteristics of FF imagery and their associations with anxiety and avoidance in SA. All participants recognized and reported on an FF image or impression of a feared catastrophe related to their SA, and FF’s were all to some extent experienced recurrently in speech situations. FF images were mostly experienced from a field perspective, and were on average rated as being vivid, distressing, and accompanied by negative feelings. Similarity of images with associated autobiographical memories was rated as moderate. Most of the associations between FF imagery characteristics and SA and avoidance behaviors were moderate to large, consistent with the expected pattern. FF image distress and the feeling that something terrible will happen showed most consistent associations with SA and avoidance measures.

The reports on recurrent experiences of FF imagery are in line with previous research demonstrating the occurrence of negative imagery in social anxiety. The content of FF images in this sample appeared to be similar to that of previously reported negative images in both clinical and non-clinical samples (e.g., descriptions of being judged negatively and performing badly; Hackmann et al., 2000; Homer and Deeprose, 2017; Ashbaugh et al., 2019). Although the present sample could be labeled as an analogue sample, the mean score on the SPS fell above the suggested clinical cut-off score of 24 (Brown et al., 1997). This indicates clinical levels of performance fears, and, therefore, may explain the high rates of reported FF imagery. Thus, findings support the idea that FF imagery occurs recurrently in (impending) speech situations in SA and reflects similar concerns as the broader variety of SAD imagery.

Furthermore, moderate to extreme vividness and distress of FF images with accompanying negative feelings also fits expectations and compares to previous findings on negative imagery in social anxiety (e.g., Schreiber and Steil, 2013; but see Moscovitch et al., 2011 for mixed findings regarding vividness in an analogue sample). Accompanying negative feelings were characterized by anxiety, shame and helplessness, to a moderate extent by sadness and the feeling that something terrible will happen, and not so much by anger and guilt. These feelings associated with imagining a catastrophic outcome link logically to SA. Sadness and helplessness could possibly also relate to heightened levels of depression, as in this sample with SA, mean CES-D scores appeared to be higher than previously found in unselected female students (Bouma et al., 1995).

Findings of more field than observer FF image perspective differed from our expectations and are more in line with studies in analogue samples that also found similar or even higher rates of field than observer perspective (Moscovitch et al., 2011; Homer and Deeprose, 2017), than with studies in SAD that found higher rates of observer perspective (Hackmann et al., 1998; Schreiber and Steil, 2013). These differences in image perspective may relate to the notion that SA concerns performance fears rather than wide-ranging social interaction and observation fears (Bögels et al., 2010). For example, individuals with SA who fear being judged negatively may predominantly see the audience and other’s judgement from their own eyes when imagining feared outcomes. Alternatively, higher rates of field perspective may also relate to closer temporal distance from the present in imagery of imminent disaster versus imagery of more distant memories (D’Argembeau and Van der Linden, 2004). In addition, as it has been indicated that individuals can experience more than one perspective during imagery, using separate scales for field and observer perspective may perhaps better represent the presence of variable perspectives (Rice and Rubin, 2009). Future research could examine whether field perspective in FF’s indeed relates more to performance fears than to the broader type of social fears.

Almost all participants were able to recall a specific autobiographical memory that was emotionally linked to their FF image but, interestingly, the similarity of both was only moderate. Previously, images in social anxiety were hypothesized to be linked to or based on childhood memories dating back to the time around the onset of the SAD (e.g., Hackmann et al., 2000). In our study, imagery about anticipated threat in SA was not always based on a specific memory of a negative speech-related event; possibly, by using an affect-bridge to assess associated memories, participants may have recalled autobiographical memories that were mainly similar regarding emotional valence, but not regarding setting, resulting in lower ratings on similarity. It is suggested that memories of multiple experiences are integrated in FF’s, including experiences in other settings than speech situations, or, although not assessed in our study, fantasy-based simulations (Rachman, 1977; Dadds et al., 1997). However, it remains difficult to reliably assess to what extent future-oriented FF images are fantasy-based versus veridical (Çili and Stopa, 2021), and thus, to categorize these FF images in SA.

The current cross-sectional associations between imagery characteristics and anxiety and avoidance provide initial support for the proposed role of FF imagery in the persistence of SA. That is, image distress, vividness, and the concomitant feeling that something terrible will happen were moderately to strongly related to global levels of SA and avoidance behaviors in speech situations, and distress and the feeling that something terrible will happen also to anticipatory anxiety and avoidance. Even though these findings need to be interpreted with caution due the correlational nature and the potentially inflated risk of type I error with multiple comparisons, some tentative inferences can be made. Imagining SA-related catastrophes more vividly and experiencing higher distress and negative feelings with such imagery may impact SA and the urge to use avoidance behaviors in speech situations. Of course, it may also work the other way around; when (anticipatory) anxiety is high, individuals may be more likely to generate or experience distressing future scenarios or to perceive danger and distress with these scenarios (Miloyan et al., 2014). Consequently, as predicted by the cognitive model of Clark and Wells (1995), the use of safety behaviors to prevent this imminent catastrophe may impede disconfirmation of beliefs and distorted imagery, on the one hand due to attribution of the non-occurrence of the catastrophic outcome to the use of safety behaviors, and on the other hand due to potential actual negative outcome because of worse performance, thereby fueling SA persistence.

Findings that image perspective and vividness were not related to anticipatory anxiety and avoidance just before the speech task, were unexpected, even though associations with global SA levels and avoidance behaviors were in the expected direction (note that image perspective was related only to avoidance behaviors). One explanation may be that the SAFE questionnaire used to assess avoidance behaviors taps in to different aspects of avoidance (i.e., safety behaviors to prevent harm while ‘enduring’ speeches) than the current anticipatory avoidance scale (i.e., tendency to completely avoid the speech; Pittig et al., 2020). Indeed, the measures were only moderately correlated, suggesting that they assess something different. Possibly, imagining oneself in catastrophic speech scenarios through others’ eyes relates to the tendency to use safety behaviors to prevent harm during speeches, but not to complete avoidance. Also, the current speech task may have been different from usual fear-provoking speeches, as also suggested by moderate correlations between the more global questionnaires and anticipatory ratings. Vividness of imagining one’s typical catastrophe may be unrelated to anticipatory anxiety or avoidance in the prospect of this speech task in a research setting. However, replications are necessary.

As the current study has confirmed the occurrence of FF imagery in SA, and has pointed to specific features and their associations with SA and avoidance, this may help to advance our knowledge on relevant imagery types and features to further examine and incorporate in treatment. As a next step, future studies that experimentally manipulate FF imagery features such as distress and vividness are essential to test their causal role in SA and avoidance. If future research corroborates the current pattern of results, it could be beneficial to incorporate this in anxiety treatment. For example, individuals with SA may be more willing to face speech situations with less safety behaviors when distressing and vivid FF images have been degraded. Thereby, the effectiveness of current treatment could possibly be enhanced. Until now, interventions that specifically target negative FF imagery have been scarcely studied in the context of treating social fears, but there have been developments regarding rescripting of memories linked to recurrent imagery in SAD and imagery-enhanced cognitive behavioral treatment for SAD (e.g., Lee and Kwon, 2013; McEvoy et al., 2018). Further research into the effects of specific interventions should aid in fine-tuning these approaches. Moreover, it would be relevant for future research to examine these imagery processes and interventions in children and adolescents to account for developmental changes in the use and content of imagery and to tailor interventions (Burnett Heyes et al., 2013).

Nevertheless, based on the current findings, we would encourage clinicians to assess imagery related to social fears, including FF imagery, as it may contain valuable information on core fears and links to current anxiety and avoidance behaviors. This could be done by, for example, incorporating a semi-structured imagery interview in the assessment (e.g., based on Hackmann et al. (2000), as in the current study), or using (explorative) imagery techniques from Schema Therapy and Metacognitive Interpersonal Therapy (e.g., Dimaggio et al., 2020; Simpson and Arntz, 2020). One could work directly with recurrent negative FF images, as these images seem to contain patients’ personally relevant worst-case scenarios constructed from varying combinations of imaginary details and memory-related details. However, findings also suggest that it may be helpful to work with associated memories of particular past events to correct distortions and associated beliefs with regard to both memories and current imagery based on new information and insights (e.g., working with an associated memory of a past bullying experience during a presentation to develop a more functional perspective on the event and associated meanings as well as a more realistic current image related to presenting; for similar approaches, also see Dimaggio et al., 2020; Simpson and Arntz, 2020).

A limitation of this study is the specific sample of female Dutch students with elevated levels of SA. As questionnaire scores indicated clinical levels of performance fears and elevated levels of depression, relevance for clinical populations increases. However, generalization to men, as well as to other countries and levels of education is restricted. Furthermore, the sample size of 60 resulted in low power for detecting small(er) effects, with the sensitivity analysis indicating a minimum effect size for correlations of 0.35 (i.e., a moderate correlation) to be reliably detected with 80% power. Considering the interview, deliberately recalled FF images were assessed and additional probes were included on worst-case scenarios; it has been suggested that spontaneously occurring (intrusive) images and deliberately generated images may be inherently different (Homer and Deeprose, 2017). Despite checking FF recurrence in speech situations, no assessment was made of frequency or interference. The interview also asked for FF’s experienced when SA is high; possibly, FF imagery also occurs at other times. Future research may improve FF imagery assessment based on these limitations, and may additionally explore and compare the features and relevance of different types of imagery (e.g., by comparing intrusive FF imagery with intrusive retrospective imagery). Also, it would be relevant to examine the relationship between negative FF imagery and verbal repetitive negative thinking, as in the current study, we did not control for the level of verbal anticipatory processing (i.e., excessively analyzing what may happen; Clark and Wells, 1995). Even though it has been found that vivid negative images are experienced during anticipatory processing (e.g., Hinrichsen and Clark, 2003; Mills et al., 2014), it is not yet clear how imagery is related to (the rate of) verbal repetitive negative thinking.

Strengths of the current study are that the social-threat induction allowed us to collect valid information on anticipatory anxiety and avoidance, compared to potentially biased retrospective reports. As the imagery interview was conducted after the speech task, this prevented the interview from influencing these anticipatory reports, since deliberate recall of negative imagery can impact subsequent anxiety, even in participants who do otherwise not spontaneously experience images in speech anticipation. Also, the task possibly increased the validity of the interview on FF imagery that participants experience in their daily lives when their SA is high, as cognitive concerns and related processes are likely to be activated after fearful events (Clark and Wells, 1995). However, it could also impose a disadvantage, as the task may have impacted reports of FF imagery, for example, based on experiences during or after the speech task.

In conclusion, it appears that recurrent vivid and distressing FF imagery is prevalent in SA, and that its characteristics are cross-sectionally associated with SA and avoidance behaviors. Performance fears may present with specific imagery characteristics such as field perspective in imagery of future threats. Thereby, findings also add to the conceptualization of SA. Findings further support the clinical relevance of assessing FF imagery in SA via, for example, a semi-structured interview, as these reports on FF images may also provide valuable information on core fears and concomitant emotional states. Associated autobiographical memories may also be relevant to assess, although FF imagery may only be moderately similar to specific associated memories, and may contain details of other memories or fantasies. If future experimental studies confirm FF imagery’s proposed causal role in SA and avoidance, interventions could be tailored to target detrimental imagery characteristics.

## Data availability statement

The data that supports the findings of this study are not publicly available because of participant privacy. Requests to access the data should be directed to the corresponding author.

## Ethics statement

The studies involving human participants were reviewed and approved by Ethics Review Committee Psychology and Neuroscience of Maastricht University 12_03_2013. The participants provided their written informed consent to participate in this study.

## Author contributions

MV: supervision of data collection. MT, MN, PJ, MR, and MV: current study conception and design considering subset of research data. MT: data analysis and first draft of the manuscript. All authors contributed to the article and approved the submitted version.

## Funding

This work was, in part, supported by a research fund awarded to MT and MN by the educational institution Post MSc Psychology and Orthopedagogics (Opleidingsinstituut PPO) linked to the University of Groningen. Opleidingsinstituut PPO had no role in the study design, data collection and analysis, decision to publish, or preparation of the manuscript.

## Conflict of interest

The authors declare that the research was conducted in the absence of any commercial or financial relationships that could be construed as a potential conflict of interest.

## Publisher’s note

All claims expressed in this article are solely those of the authors and do not necessarily represent those of their affiliated organizations, or those of the publisher, the editors and the reviewers. Any product that may be evaluated in this article, or claim that may be made by its manufacturer, is not guaranteed or endorsed by the publisher.
